# Local Spin Density Approximation Strongly Improved by a Better-Informed Local Scaling of Its Self-Interaction Correction

**DOI:** 10.1021/acs.jctc.6c00357

**Published:** 2026-05-29

**Authors:** Chandra Shahi, Rohan Maniar, Jinliang Ning, Raj K. Sah, Mark R. Pederson, Adrienn Ruzsinszky, Juan E. Peralta, Koblar A. Jackson, John P. Perdew

**Affiliations:** † Department of Physics and Engineering Physics, 5783 Tulane University, New Orleans, Louisiana 70118, United States; ‡ Department of Physics, 6558 Temple University, Philadelphia, Pennsylvania 19122, United States; § Department of Physics, University of Texas at El Paso, El Paso, Texas 79968, United States; ∥ Department of Physics, 5649 Central Michigan University, Mount Pleasant, Michigan 48859, United States

## Abstract

The Perdew–Zunger self-interaction correction (PZSIC) makes density functional approximations (DFAs) exact for all one-electron densities. However, it overcorrects in many-electron regions, introducing errors for the uniform-density limit, where uncorrected DFAs are exact. The locally scaled PZSIC (LSIC), based on the iso-orbital indicator *z*_σ_ [which distinguishes single-orbital and slowly varying density regions and is used with the local spin density approximation (LSDA)], restores the uniform-density limit and significantly improves results for many properties, including chemical reaction barrier heights, atomization energies, and ionization potentials. Yet, LSIC performs poorly for weakly bonded systems, leaving many unbound, due to limitations of its iso-orbital indicator. To correct this, in this work we propose a new local scaling, LSIC-α, based on the iso-orbital indicator α_σ_ (which additionally identifies regions of overlapping density tails). A two-parameter scaling function of α_σ_ is fitted to a subset of the nonbonded appropriate norms for the SCAN and *r* ^2^SCAN meta-GGAs, and tested on many properties of main-group atoms, molecules, and molecular complexes. LSIC-α greatly improves the interaction energies of weakly bonded systems in the S22 data set while retaining LSIC’s accuracy for other properties. This work shows that the errors of LSDA (and presumably of higher-level DFAs) can be largely but not entirely repaired by a proper “do no harm” self-interaction correction.

## Graphical abstract

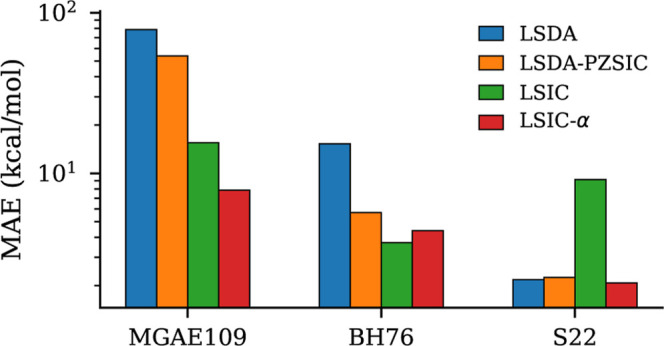

## Introduction

In Kohn–Sham density functional theory (DFT),^1,2^ the exchange–correlation energy, which encapsulates the complex many-body effects of electron–electron interactions, must be approximated to make the method computationally tractable for atoms, molecules, and condensed-phase systems. Within the semilocal approximation, the exchange–correlation energy functional takes the form of a single spatial integral over an energy density, as shown in eq 1

$$E_{xc}\left[n_{↑},n_{↓}\right]=\int d^{3}r{n\varepsilon }_{xc}\left(n_{↑},n_{↓},\nabla n_{↑},\nabla n_{↓},\tau_{↑},\tau_{↓}\right)$$ 1

Here, $n_{\sigma }\left(r\right)=\sum_{i}^{occ}|\psi_{i\sigma }{|}^{2}\left(with\sigma =↑,↓\right)$ is the spin-resolved electron density, ∇*n*_σ_(**r**) is its gradient, *n*(**r**) = ∑_σ_ *n*_σ_(**r**), and $\tau_{\sigma }\left(r\right)=\frac{1}{2}\sum_{i}^{occ}|\nabla \psi_{i\sigma }\left(r\right){|}^{2}$ is the kinetic energy density constructed from the occupied Kohn–Sham (KS) orbitals of spin σ, ψ_*i*σ_.

In the local spin density approximation (LSDA),^2,3^ the exchange–correlation energy density per electron ε_*xc*_ depends only on the local spin densities *n*_↑_(**r**) and *n*_↓_(**r**) at position **r**, under the assumption of a locally uniform electron gas. This simple approximation is much better than it was initially expected to be, because it implicitly satisfies several exact constraints on the exact exchange–correlation hole^4,5^ and energy.^6^

To capture effects beyond the uniform electron gas model, the generalized gradient approximation (GGA) incorporates the dependence of the exchange–correlation energy on the density gradients ∇*n*_σ_. Meta-GGA functionals further extend this framework by including additional ingredients such as the kinetic energy density τ_σ_ and, in some cases, the Laplacian of the density.

More accurate and versatile density functional approximations (DFAs) are developed by enforcing known mathematical properties—referred to as exact constraints that the exchange–correlation energy should satisfy. In general, the predictive power of a DFA improves with the number of exact constraints it satisfies,^6^ as evidenced by the increased accuracy from LSDA to the Perdew–Burke–Ernzerhof (PBE^7^) GGA, and then to the strongly constrained and appropriately normed (SCAN^8^) meta-GGA functionals, and its regularized-restored version *r* ^2^SCAN.^9^

One such constraint^6,10^ is that, for any one-electron system with density *n*_*i*σ_, the sum of the exchange–correlation energy and the classical Hartree energy must vanish, as in eq 2

2 $$E_{xc}\left[n_{i\sigma },0\right]+U\left[n_{i\sigma }\right]=0$$

where the classical Hartree self-energy is defined in eq 3

3 $$U\left[n_{i\sigma }\right]=\frac{1}{2}\iint d^{3}rd^{3}r^{\prime }\frac{n_{i\sigma }\left(r\right)n_{i\sigma }\left(r^{\prime }\right)}{\left|r-r^{\prime }\right|}$$

However, all local and semilocal (LSDA, GGA, and meta-GGA) functionals are known to violate this fundamental condition, leading to the so-called self-interaction error (SIE). The SIE becomes particularly pronounced in systems where the electron density is delocalized over stretched bonds, as in the transition state of a chemical reaction. For example, in H_2_^+^, ref 11 demonstrates that semilocal DFAs such as PBE and SCAN exhibit negligible SIE when the electron density is compact, but the error increases sharply as the bond is stretched. Local and semilocal DFAs force the exchange–correlation hole to remain localized near the reference electron, while the exact hole density in a one-electron density *n*_σ_(**r**) is −*n*_σ_(**r**), independent of the electron’s position and delocalized over the whole system. Thus, the approximated exchange–correlation energy—and therefore the total energy—becomes too negative. This overbinding leads to the systematic underestimation of barriers for chemical reactions by local and semilocal DFAs. Other important consequences of SIE in DFAs include overly high energies of the highest occupied molecular orbital (HOMO) and the spurious destabilization of small anionic species.^12^

Although several approaches^13–17^ have been proposed to mitigate SIE, the Perdew–Zunger self-interaction correction (PZSIC)^10^ eliminates it completely from any DFA. In this method, the one-electron self-interaction error, represented by the left-hand side of eq 2, is removed on an orbital-by-orbital basis. The corrected exchange–correlation energy is given by eq 4

4 $$E_{xc}^{PZSIC}=E_{xc}^{DFA}\left[n_{↑},n_{↓}\right]-\sum_{i\sigma }\left(E_{xc}^{DFA}\left[n_{i\sigma },0\right]+U\left[n_{i\sigma }\right]\right)$$

For size-consistency, the orbitals whose densities appear in eq 4 should be localized and unitarily equivalent to the occupied KS orbitals.^10,18–21^

Thus, enforcing the exact condition of eq 2 through PZSIC renders DFAs free of self-interaction error for any one-electron density, leading to improved performance in systems where SIE is pronounced. However, when applied to semilocal DFAs, PZSIC often degrades equilibrium properties^11,22,23^ such as atomization energies and bond lengths—quantities for which the uncorrected semilocal functionals, particularly SCAN, already achieve high accuracy.

This degradation of performance occurs because the PZSIC is being applied not only to one-electron regions but also to many-electron regions where the DFA may need less or no correction. An example is the uniform-density limit, for which the uncorrected DFAs are exact. It was recognized early on that PZSIC tends to overcorrect in many-electron regions, creating the need to scale down the correction.^24–26^ This was accomplished by scaling the PZ correction either globally^27,28^ or on an orbital-by-orbital basis.^25^ The simplest scaling^27,28^ by a global factor of 0.5 is a good average for many molecules, but is not exact for either one-electron densities or the uniform density limit.

A recent work by Santra and Perdew^29^ provided a quantitative analysis for the uniform density limit, showing that, in the uniform-density limit, PZSIC with real localized orbitals applied to LSDA yields an error of approximately +5.5% in the exchange–correlation energy, while application to PBE and SCAN produces errors of about −3.5%.

The uniform-density limit can be restored by scaling down the PZSIC energy density. A recently developed locally scaled PZSIC for LSDA, referred to as LSIC,^30^ employs internal scaling—that is, point-by-point scaling—of the SIC energy density. LSIC provides remarkable improvement over both LSDA and LSDA with unscaled PZSIC, and it avoids the gauge inconsistency^31^ between the Hartree and exchange–correlation energy densities observed in PBE and SCAN. However, LSIC performs poorly for weakly bonded systems.^32^ In this work, we propose a new local scaling method, termed LSIC-α, which addresses LSIC’s deficiencies for weak bonds while preserving its high accuracy for other properties.

### Theory and Computational Details

The internally scaled form of the PZSIC applied to LSDA exchange–correlation energy can be written as eq 5

$$E_{xc}^{LSIC}=E_{xc}^{LSDA}\left[n_{↑},n_{↓}\right]-\overset{occ}{\underset{i\sigma }{\sum }}\int d^{3}r\;n_{i\sigma }\left(r\right)f_{\sigma }\left(r\right)\times \left[\frac{1}{2}u(\left[n_{i\sigma }];r\right)+\varepsilon_{xc}^{LSDA}\left(n_{i\sigma }(r),0\right)\right],$$ 5

where ε_*xc*_^LSDA^(*n*↑, *n*↓) is the exchange-correlation energy density per electron in an electron gas with uniform spin densities (*n*↑, *n*↓), and the Hartree self-interaction potential of the orbital density *n*_*i*σ_ is defined in eq 6

$$u\left([n_{i\sigma }];r\right)=\int d^{3}r^{\prime }\frac{n_{i\sigma }\left(r^{\prime }\right)}{\left|r-r^{\prime }\right|}.$$ 6

For LSIC based on LSDA, the scaling function *f*_σ_ is chosen to be a function of the iso-orbital indicator *z*_σ_, as defined in eq 7

7 $$f_{\sigma }\left(r\right)=z_{\sigma }=\frac{\tau_{\sigma }^{W}}{\tau_{\sigma }}$$

where $\tau_{\sigma }^{W}=\frac{|\nabla n_{\sigma }{|}^{2}}{8n_{\sigma }}$ is the von Weizsäcker kinetic energy density, corresponding to the single-orbital limit of τ_σ_ = 1/2∑_*i*σ_|∇ψ_*i*σ_|^2^.

In regions where τ_σ_(**r**) = τ_σ_^W^(**r**) (i.e., *z*_σ_ = 1), the density is single-electron-like and LSIC recovers the full PZSIC. In the opposite limit, τ_σ_^W^(**r**) = 0 (*z*_σ_ = 0), corresponding to a uniform density or many-electron region, LSIC reduces to LSDA, thereby eliminating the spurious PZSIC contribution. Since 0 ≤ *z*_σ_ ≤ 1, LSIC provides a smooth and parameter-free interpolation between LSDA and LSDA-PZSIC.

The iso-orbital indicator *z*_σ_ can distinguish single-electron-like regions from many-electron regions. However, in iso-orbital regions where ∇*n*_σ_ → 0, such as the bond center of a covalent bond, the limiting value of *z* depends on the order in which the iso-orbital limit and the slowly varying density limit are taken, leading to an order-of-limits problem.^33^ In weakly bonded systems, an additional issue arises.^32^ At the bond center, where the density tail of an orbital with spin σ overlaps with the tail of a different orbital of the same spin, *z*_σ_ ≈ 0 (τ_σ_ ≫ τ_σ_^W^), so no PZSIC is applied. In contrast, in the low-density tail region of an orbital with spin σ, *z*_σ_ = 1, and full PZSIC is applied (see Figures S2 and S3.). This mismatch in the treatment of overlapping density tails versus isolated tails leads to an inconsistent and often inaccurate description of weak bonds in LSIC.

To address the limitations of *z*_σ_, we introduce in LSIC-α a new scale-down factor *f* in eq 8

$$f_{\sigma }\left(r\right)=p\left(a,b,\alpha_{\sigma }\right)={\left(\frac{{\left(1-\alpha_{\sigma }\right)}^{2}}{1-a\alpha_{\sigma }+\alpha_{\sigma }^{2}}\right)}^{\frac{\alpha_{\sigma }\left(1+b\alpha_{\sigma }\right)}{\left(b+\alpha_{\sigma }^{2}\right)}},$$ 8

based on an alternative non-negative iso-orbital indicator used in SCAN and defined in eq 9

9 $$\alpha_{\sigma }=\frac{\tau_{\sigma }-\tau_{\sigma }^{W}}{\tau_{\sigma }^{unif}},$$

where $\tau_{\sigma }^{unif}=\frac{3}{10}{\left(6\pi^{2}\right)}^{2/3}n_{\sigma }^{5/3}$ is the uniform-density limit of τ_σ_. A possible three-parameter generalization of eq 8 is discussed in the Supporting Information and illustrated in Figure S6.

The outermost exponent in eq 8 varies from 0 to 1 to *b* as α_σ_ varies from 0 to 1 to ∞. To keep *f*_σ_ bounded between 0 and 1 requires *a* ≤ 2 and *b* > 0. *a* = 2 makes *f*_σ_ = 1 and recovers LSDA-PZSIC, while *a* → – *∞* makes *f*_σ_ → 0 and recovers LSDA. The parameters *a* = 1.829 and *b* = 21.31 were determined by fitting to the exchange–correlation energies of rare gas atoms (Ne, Ar, Kr, and Xe) and the interaction energies of compressed Ar_2_ at bond lengths *R* = 1.6, 1.8, and 2.0 Å. These were also appropriate norms for SCAN. Since all the norms of SCAN/*r* ^2^SCAN and LSIC-α are nonbonded, the results of those functionals for bond properties are genuine predictions. The goal of LSIC-α is a self-interaction correction to LSDA that does no harm. The LSIC-α norm errors are reported in Tables S1 and S2 of the Supporting Information, where they may be compared with those of *r* ^2^SCAN.

The indicator α_σ_(**r**) can distinguish^8^ between single-orbital regions (α_σ_ = 0), slowly varying metallic densities (α_σ_ ≈ 1), and the slowly varying density-overlap regions of weak bonds (α_σ_ ≫ 1), which *z*_σ_ cannot. Figure 1 illustrates the scale-down function *f*(α) used in LSIC-α. It approaches unity at α = 0 (single-orbital regions), vanishes near α ≈ 1 (slowly varying densities), and approaches unity again for α ≫ 1. In asymptotic density tails where *z*_σ_ tends to 1, and where full SIC is needed, α_σ_ tends to either 0 (for *s*-like Frontier orbitals) or ∞. LSIC-α has the following properties: it recovers full PZSIC for both α_σ_ = 0 and α ≫ 1, and provides no correction to LSDA for α_σ_ = 1. Furthermore, in the slowly varying density limit (α_σ_ ≈ 1), where α_σ_ = 1 + O­(∇^2^) and *z*_σ_ = O­(∇^2^), LSIC-α reduces to LSDA for slowly varying nonuniform densities. In contrast, LSIC reduces to LSDA only in the uniform-density limit, leaving a spuriously large second-order gradient correction to LSDA. Figures S1 and S2 show the behavior of α_σ_ = α and *z*_σ_ = *z* for the Ar atom and Ar_2_ dimer, respectively, along the radial and internuclear axes, illustrating the distinct behavior of the two indicators in atomic and weak-bond overlap regions. Figure S3 further compares the corresponding LSIC and LSIC-α scale-down factors for Ar_2_, showing their different behavior in the weak density-overlap region.

**Figure 1:**
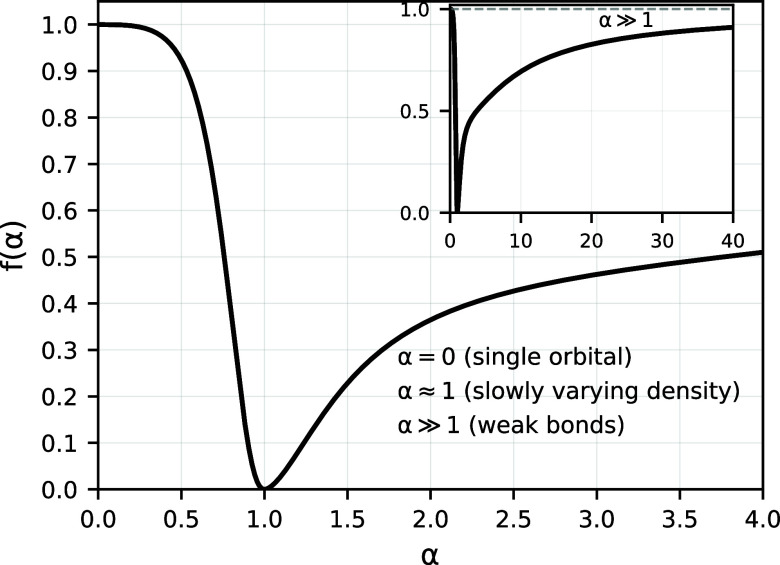
Scale-down function *f*(α) used in the LSIC-α version of eq 5. The function smoothly interpolates between LSDA and PZSIC, approaching unity at α = 0 and α ≫ 1, while vanishing near α ≈ 1. The inset shows the large-α behavior.

We implemented LSIC-α in the Fermi–Löwdin orbital self-interaction correction (FLOSIC) code.^34^ The FLOSIC method^19^ is a unitarily invariant implementation of PZSIC that uses localized Fermi orbitals defined in eq 10

10 $$F_{i\sigma }\left(r\right)=\sum_{j}^{occ}\frac{\psi_{j\sigma }\left(r\right)\psi_{j\sigma }\left(a_{i\sigma }\right)}{\sqrt{n_{\sigma }\left(a_{i\sigma }\right)}},$$

constructed from the occupied KS orbitals. These are further symmetrically orthogonalized to obtain a set of SIC orbitals, ϕ_*i*σ_, called Fermi–Löwdin orbitals, that are used in evaluating the SIC energy. This is a special unitary transformation of the occupied KS orbitals that makes the orbital density $n_{i\sigma }\left(r\right)={\left|\phi_{i\sigma }\left(r\right)\right|}^{2}$ in eqs 4 and 5 localized: ${\left|F_{i\sigma }\left(a_{i\sigma }\right)\right|}^{2}=\sum_{j}^{occ}{\left|\psi_{j\sigma }\left(a_{i\sigma }\right)\right|}^{2}=n_{\sigma }\left(a_{i\sigma }\right)$ . The Fermi-orbital descriptor (FODs) **a**_**iσ**_ are positions in space that are found by minimizing the self-interaction-corrected energy.

We used the fodMC code^35^ to generate the initial FODs and then computed LSDA-PZSIC energies by optimizing the FOD positions until the magnitude of the energy gradients with respect to the FOD coordinates dropped below 5× 10^–4^ Ha/bohr. The SCF tolerance was set to 10^–6^ Ha, and the NRLMOL basis set^36^ was used. The LSIC-α energies were evaluated nonself-consistently using the converged LSDA-PZSIC orbitals and FOD positions. LSIC-α energies and energy differences found in this way should be very close to those of self-consistent LSIC-α, as found earlier^37^ for LSIC. That is presumably because significant density-driven errors of the energy in normally correlated systems arise only from electron-transfer errors^38^ that are corrected by SIC. All calculations were carried out using the fine integration grids routinely employed in FLOSIC calculations, corresponding to MESHSETTING = 1. The LSIC-α Ar_2_ potential–energy curve, see Figure S4, is smooth, and we therefore expect LSIC-α to be no more grid-sensitive than standard FLOSIC calculations.

Recently, the original LSIC has been generalized to complex orbitals and implemented^39^ self-consistently in the GPAW code^40^ as a correction to the PBE GGA.

## Results and Discussion

We evaluate the performance of LSIC-α on several benchmark data sets commonly used in computational chemistry to assess the accuracy of exchange–correlation methods. For comparison, we include results from LSDA, LSDA-PZSIC, LSIC, and the semilocal PBE and *r* ^2^SCAN functionals. Because LSDA is derived from the uniform electron gas model, it is generally expected to perform reasonably well in systems with slowly varying densities, but it can fail in molecular systems, where the density changes rapidly. Overall, LSIC-α yields significant improvement over LSDA and LSDA-PZSIC, and outperforms PBE in many cases. Figure 2 compares the MAEs of LSDA, LSDA-PZSIC, LSIC, and LSIC-α across the eight data sets. Complete numerical results for all eight data sets are reported in the Supporting Information.

**Figure 2:**
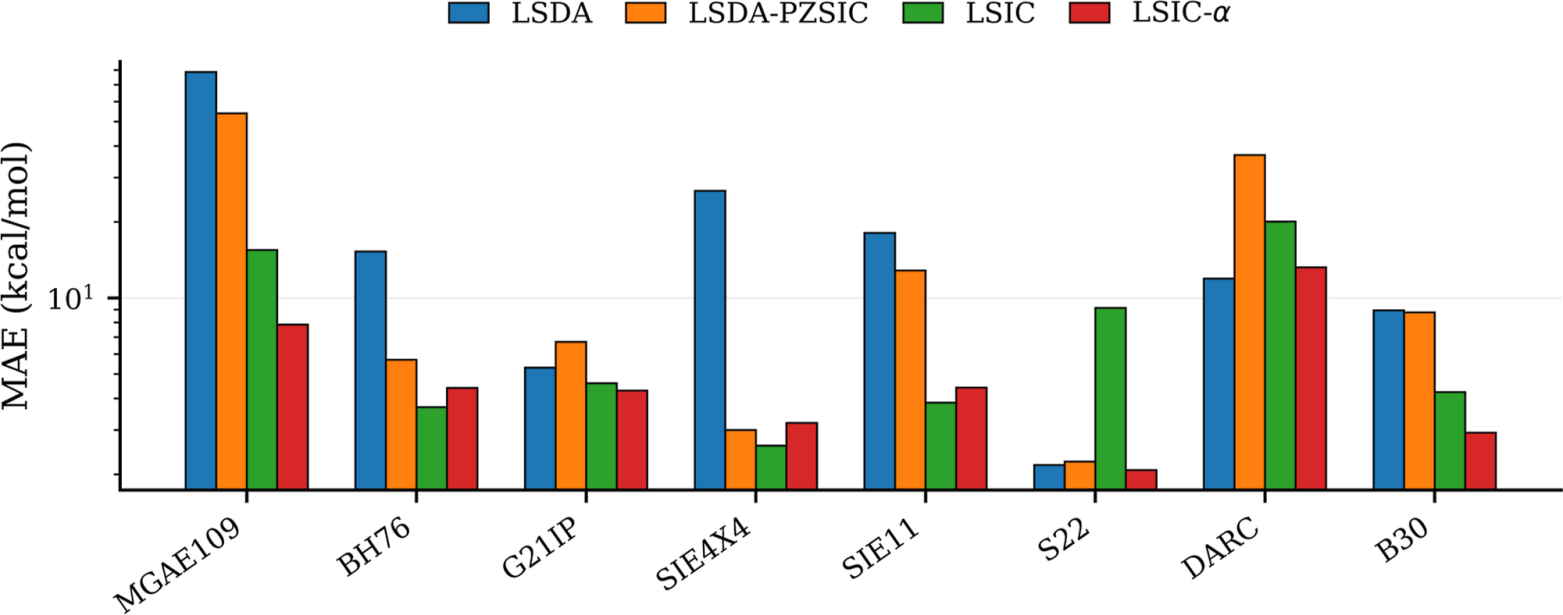
Mean absolute errors (MAEs) of LSDA, LSDA-PZSIC, LSIC, and LSIC-α across the eight benchmark data sets (logarithmic scale).

We begin with the main-group atomization energies from the MGAE109 data set,^41^ which provide a stringent test of the balance between atomic and molecular total energies. Table 1 presents the mean absolute errors (MAEs) for the different methods. LSDA performs the worst, with an MAE of 78.69 kcal/mol, because it overbinds molecules by making the separated atoms too high in energy relative to the molecule, highlighting the large SIE error of LSDA for the compact densities of atoms. A similar error of LSDA (−10% of the negative exchange–correlation energy) for the compact density of He^+^ can be observed^11^ in the H_2_^+^ molecule at *R* = 0 bohr. Applying PZSIC to LSDA improves the atomization energies, reducing the MAE to 53.94 kcal/mol. However, when PZSIC is applied to more realistic semilocal functionals like PBE or SCAN, it generally worsens their good behavior. By contrast, LSIC-α dramatically reduces the MGAE109 atomization energy errors to 7.85 kcal/mol, outperforming both LSIC (12.51 kcal/mol) and PBE (13.17 kcal/mol). The improved performance of LSIC-α for atomization energies arises from the nonuniform effect of the local scaling on atoms and molecules. As shown in Figure S5, LSIC-α retains a larger fraction of the full PZSIC correction for atoms than for molecules. This reflects the greater prevalence of slowly varying density regions in molecules compared to atoms, where the scaling reduces the self-interaction correction. As a result, LSIC-α lowers atomic energies more than molecular energies relative to LSDA, leading to a systematic reduction of the overbinding present in LSDA and LSDA-PZSIC.

**Table 1:** Mean Error (ME) and Mean Absolute Error (MAE) in kcal/mol for the Atomization Energies of the MGAE109 Dataset, Defined as *E*_atomization_ = ∑_*i*_ *E*_atom,*i*_—*E*_molecule_ ^a^

method	ME	MAE
LSDA	78.69	78.69
LSDA-PZSIC	52.04	53.94
LSIC	–4.27	12.51
LSIC-α	–0.89	7.85
PBE	11.44	13.17
*r* ^2^SCAN	–0.03	3.13

a Reference geometries and benchmark atomization energies were taken from ref 41. The mean value of the reference atomization energies is 498.53 kcal/mol. (1 kcal/mol = 0.0456 eV).

Having established the improvement in atomization energies, we next examine reaction barrier heights for chemical reactions, which probe stretched bonds of transition states. Table 2 summarizes the errors for the BH76 data set.^42^ Because transition states involve stretched (hence noncompact) bonds, semilocal DFAs tend to predict transition-state energies that are too low relative to the reactants, thereby underestimating the barriers. Across both uncorrected DFAs and their PZSIC variants,^11^ accuracy improves in the order LSDA < PBE < *r* ^2^SCAN. LSIC (MAE = 1.3 kcal/mol)^30^ yields remarkably accurate barriers for the smaller BH6 data set,^43^ while LSIC-α (MAE = 3.0 kcal/mol) is slightly worse but comparable to SCAN-PZSIC^11^ (MAE = 3.0 kcal/mol). On the larger BH76 set,^42^ LSIC (3.7 kcal/mol) and LSIC-α (4.4 kcal/mol) show comparable accuracy, confirming the robustness of both approaches for barrier heights.

**Table 2:** Mean Error (ME) and Mean Absolute Error (MAE) in kcal/mol for the Barrier Heights of the BH76 Dataset, Defined as the Energy Difference between the Transition State and the Reactants. Reference Geometries and Benchmark Barrier Heights were Taken from Ref 42 ^a^

method	ME	MAE
LSDA	–15.2	15.3
LSDA-PZSIC	–0.2	5.7
LSIC	0.7	3.7
LSIC-α	0.3	4.4
PBE	–9.4	9.4
*r* ^2^SCAN	–7.3	7.3

a The mean and mean absolute values of the reference barrier heights are 18.2 and 18.6 kcal/mol, respectively.

Ionization potentials (IPs) are also highly sensitive to self-interaction error, as they involve the removal of an electron from the outermost occupied orbital. In semilocal density functional approximations, the exchange–correlation potential decays too rapidly compared to the exact asymptotic behavior, which follows a −1/*r* form. As a consequence, the HOMO energies are too high, leading to systematic underestimation of IPs. The first vertical ionization potential should equal^44^ the negative of the HOMO energy. Previous work^45^ has shown that PZSIC improves LSDA HOMO energies by reducing self-interaction error. In the present work, however, we compute adiabatic (not vertical) ionization potentials using the ΔSCF approach, defined as the total energy difference between the cationic and neutral systems, each evaluated at its relaxed geometry. Table 3 summarizes the IP errors for the G21IP data set.^42^ While PZSIC worsens the LSDA IPs, both locally scaled variants significantly improve upon PZSIC and also reduce the errors relative to LSDA.

**Table 3:** Mean Error (ME) and Mean Absolute Error (MAE) in kcal/mol for the ΔSCF Adiabatic First IPs of the G21IP Dataset^a^

method	ME	MAE
LSDA	3.1	5.3
LSDA-PZSIC	6.0	6.7
LSIC	2.7	4.6
LSIC-α	0.1	4.3
PBE	–0.1	4.1
*r* ^2^SCAN	–0.2	4.5

a Reference geometries and benchmark IPs were taken from ref 42. The mean value of the reference IPs is 257.6 kcal/mol.

Self-interaction error (SIE) test sets are explicitly designed to probe the magnitude of the SIE in density functional approximations. (The SIE4 × 4 set includes the binding energies of 4 positively charged dimers, each at four different bond lengths. The SIE11 set includes 11 positively charged or neutral molecules whose energies are extremely prone to self-interaction error.) Table 4 shows that local and semilocal DFAs exhibit substantial errors in the binding energies of the SIE4 × 4 data set and the reaction energies of the SIE11 data set. Applying PZSIC significantly reduces these errors,^46^ while the locally scaled variants further improve the results for the SIE11 set.^46^

**Table 4:** Mean Absolute Errors (MAEs) in kcal/mol for the SIE4 × 4 and SIE11 Datasets, Reference Geometries and Benchmark Binding and Reaction Energies were Taken from refs 42 and 47 ,^a^

method	SIE4 × 4	SIE11
LSDA	26.6	18.13
LSDA-PZSIC	3.0	9.70
LSIC	2.6	3.85
LSIC-α	3.2	4.42
PBE	23.3	12.03
*r* ^2^SCAN	18.0	9.94

a The mean values of the reference values for SIE4 × 4 and SIE11 are 33.7 and 33.81 kcal/mol.

Reference 48 discussed the DARC set of 14 energies of Diels–Alder reactions, which challenge DFT by the formation of pairs of close but nonbonded carbon atoms. Table 5 shows that LSDA, PZSIC, and LSIC-α all overbind the reaction products, while LSIC strongly underbinds. Among the SIC-based methods, LSIC-α performs substantially better than both LSDA–PZSIC and LSIC, but slightly worse than LSDA. To put this into perspective, a comparison with Table 1 of ref 48 shows that the standard functionals for chemistry (which are usually much more accurate than LSDA for molecules) all overbind, with the MAE of the BLYP GGA much greater than that of LSDA, and the MAE of the B3LYP hybrid slightly greater than that of LSDA. On the other hand, *r* ^2^SCAN, which captures intermediate-range van der Waals interaction, provides much better reaction energies. Much better results^49^ for the DARC reactions are also found from the much more expensive random phase approximation, which has exact exchange and dispersion interaction.

**Table 5:** Mean Error (ME) and Mean Absolute Error (MAE) in kcal/mol for the Reaction Energies of the DARC Dataset, Reference Geometries and Benchmark Reaction Energies were Taken from ref 42,^a^

method	ME	MAE
LSDA	–11.94	11.94
LSDA-PZSIC	–36.86	36.86
LSIC	20.10	20.10
LSIC-α	–13.25	13.25
PBE	6.30	6.51
*r* ^2^SCAN	2.46	3.26

a The mean value of the reference reaction energies is −32.5 kcal/mol.

The S22 data set^50^ consists of weakly bound (hydrogen-bonded, dispersion-dominated, and mixed) complexes for which LSIC has previously been shown to perform poorly.^32^ The interaction energy of a complex was defined as

$$E_{\mathrm{int}}=E_{AB}-\left(E_{A}^{*}+E_{B}^{*}\right)$$

where *E*_AB_ is the total energy of the complex at the equilibrium geometry, and *E*_A_^*^ and *E*_B_^*^ are the total energies of the individual monomers in the distorted geometries they adopt within the complex. Table 6 summarizes the mean errors and mean absolute errors for the S22 data set. LSDA yields reasonably accurate but consistently overbinding interaction energies. Unlike in the case of atomization energies (AE6), PZSIC provides comparable estimates of the interaction energies, because its contributions in the low-density tail regions of isolated monomers and in the overlapping tail regions of the complexes approximately cancel.^32^ This cancellation does not occur in LSIC, since *z*_σ_ vanishes near the bond center, in contrast to its approach to unity in the density tail of a monomer. As a result, LSIC strongly underbinds, leaving many complexes unbound. LSIC-α restores this cancellation, as it provides nearly full PZSIC corrections in both regions, thereby improving the description of weak interactions.

**Table 6:** Mean Error (ME) and Mean Absolute Error (MAE) in kcal/mol for the Interaction Energies of the S22 Dataset^a^

method	ME	MAE
LSDA	–2.06	2.18
LSDA-PZSIC	–1.54	2.25
LSIC	9.14	9.14
LSIC-α	0.12	2.08
PBE	3.24	3.25
*r* ^2^SCAN	0.65	1.17

a Reference geometries and benchmark interaction energies were taken from ref 50. The mean of the reference interaction energies is −7.36 kcal/mol.

The MEs in Table 6 show that LSDA and LSDA-PZSIC overbind the weak bonds, while the other functionals in Table 6 underbind them. Since all these functionals omit the long-range van der Waals correction, they should all ideally underbind. The underbinding is small for LSIC-α, suggesting that it, like *r* ^2^SCAN, accounts for the intermediate-range (but not the long-range) van der Waals interaction. Dispersion effects are also significant in the B30 set to be discussed next.

The B30 data set^51^ probes noncovalent interactions beyond hydrogen bonding, including halogen-, chalcogen-, and pnictogen-bonded complexes. Table 7 shows that DFAs exhibit substantial errors for halogen-bonded systems, even for advanced functionals such as PBE and *r* ^2^SCAN, with MAEs of about 7 kcal/mol. This reflects the well-known difficulty of semilocal DFAs in describing halogen bonding,^38^ which is characterized by strongly anisotropic charge distributions and directionally dependent electrostatic interactions arising from the σ-hole on the halogen atom. Previous work^38^ has shown that applying the PZSIC to PBE improves halogen-bond interaction energies, reducing the MAE to 3.57 kcal/mol. LSIC-α yields the lowest MAE among all methods considered for halogen-bonded complexes, with an MAE of 3.94 kcal/mol. Moreover, LSIC-α performs consistently better than LSDA, LSDA-PZSIC, and LSIC across all three classes of interactions in the B30 data set.

**Table 7:** Mean Absolute Errors (MAEs) in Kcal/Mol for Interaction Energies of Halogen-, Chalcogen-, and Pnictogen-Bonded Complexes in the B30 Dataset, Computed Relative to High-Level Reference Values^53^ ^,^ ^a^

	halogen	chalcogen	pnictogen
LSDA	15.82	7.48	6.46
LSDA-PZSIC	10.72	8.41	8.47
LSIC	4.86	3.34	6.31
LSIC-α	3.94	2.37	3.58
PBE	7.09	2.53	1.01
*r* ^2^SCAN	7.02	2.12	1.13

a The mean values of reference interaction energies for the halogen-, chalcogen-, and pnictogen-bonded systems are respectively −34.12, −13.83, and −18.82 kcal/mol.

The MVO-10 benchmark data set^52^ of gas-phase mixed-valence oxides would be an interesting test for a fully self-consistent LSIC-α (not yet implemented). This data set measures how well a density functional approximation can describe both localized and delocalized electronic states.

## Conclusions

We have developed LSIC-α, a generalized local-scaling self-interaction correction to LSDA that employs the α_σ_ iso-orbital indicator and a new analytic scaling function to address known deficiencies of LSIC in weakly bonded systems. The method preserves desirable physical limits: it recovers full PZSIC in single-orbital and large-α regimes, eliminates spurious gradient corrections in the slowly varying density limit, and smoothly interpolates between LSDA and LSDA-PZSIC in a physically motivated manner. LSIC-α was benchmarked within the FLOSIC framework using the self-consistent LSDA-PZSIC orbitals and density.

Across a broad range of benchmark data sets probing atomization energies MGAE109, reaction barrier heights BH76, ionization potentials G21IP, explicit self-interaction error test sets SIE4 × 4 and SIE11, weak interactions S22, delocalization–sensitive reaction energies DARC, and anisotropic noncovalent complexes B30, LSIC-α delivers balanced and reliable performance. It systematically improves upon LSDA and LSDA-PZSIC and significantly reduces the large underbinding errors of LSIC for weak interactions, lowering S22 MAE by more than a factor of 4. For atomization energies, LSIC-α further reduces the already improved LSIC errors by over 37%. These results demonstrate that LSIC-α is a broadly transferable SIC framework, capable of reliably describing diverse bonding situations with improved accuracy over existing SIC approaches.

It is known^54^ that the exact energy of an open system with a noninteger average electron number *N* varies linearly with *N* between adjacent integers, and has derivative discontinuities at the integers. Semilocal density functional approximations can be accurate at the integers, but miss the derivative discontinuities and thus curve upward between adjacent integers, leading to a serious delocalization error with incorrectly fractional electron numbers for the dissociation products of most heteronuclear dimers, and to density-driven errors of the energy arising from electron-transfer error.^38^ Full PZSIC and LSIC^55^ both strongly reduce these errors, and the same could be expected from LSIC-α. A self-consistent implementation of LSIC-α would be needed for a quantitative test.

This work demonstrates that the largest error (but not all of the error) of LSDA can be regarded as self-interaction error, and corrected by a “do no harm” self-interaction correction. Presumably, the same is true of the higher-level PBE and SCAN functionals (although SIE decreases from LSDA to PBE to SCAN). However, as realized earlier for LSIC,^30,31^ locally scaling the sum of the self-Hartree and self-exchange-correlation energy densities works only for LSDA, because only in LSDA is the self-xc energy density in the same gauge as the self-Hartree energy density. In PBE and SCAN, the self-xc energy density has been transformed via a simplifying integration by parts. Other possibilities for a “do no harm” self-interaction corrections to PBE and SCAN exist and are under investigation.

Next to exactness for all uniform densities, exactness for all one-electron densities is the most obvious and important exact constraint on the density functional for the exchange–correlation energy, and at the same time one of the least tractable. A proper self-interaction correction that does both without doing serious harm to SCAN or *r* ^2^SCAN might rectify the systems and properties for which *r* ^2^SCAN needs a fitted + *U* or + *V* correction^56,57^ or a hybrid correction.^14,58^ We propose to include the proper self-interaction corrections on the fourth rung of Jacob’s Ladder^59^ of density functional approximations, along with the hybrid functionals. Both types are fully nonlocal functionals of the noninteracting one-particle density matrix or occupied orbitals.

The focus of this article has been on improving the accuracy of self-interaction correction. But computational efficiency is also important. Self-interaction correction is typically much more expensive than the semilocal functional it corrects, because of the need to iteratively determine the unitary transformation of the orbitals (which in FLOSIC becomes the iterative determination of the FODs in eq 10). But there is evidence that good approximations to the FODs can be constructed directly from the noninteracting one-particle density matrix or from the occupied orbitals.^60^ Thus, in a self-consistent implementation of a locally scaled-down SIC (as found for LSIC in ref 37), it should be possible to choose the parameters of the scaling function (e.g., our *a* and *b*) that work best for an inexpensive choice of the method used to find the FODs.

## Supplementary Material



## Notes

LSIC-α norm errors for rare-gas atoms; LSIC-α norm errors for compressed Ar_2_; iso-orbital indicators α and *z* for the Ar atom; iso-orbital indicators α and *z* for the Ar_2_ dimer; LSIC and LSIC-α scale-down functions of Ar_2_; Ar_2_ binding-energy curve; fraction of the PZSIC total-energy correction retained by LSIC-α for atoms and molecules; MGAE109 atomization energies for different methods; BH76 barrier heights; G21IP adiabatic ionization potentials; SIE4 × 4 self-interaction-error benchmark results; SIE11 self-interaction-error benchmark results; DARC reaction energies; S22 interaction energies; B30 interaction energies; and three parameters scale-down factor (PDF)

## |

Xinjiang Technical Institute of Physics and Chemistry, Chinese Academy of Sciences, Urumqi 830011, China

## ⊥

Tarrant County College South Campus, Fort Worth, Texas 76119, USA.
