# Supplementary material for: Local Spin Density Approximation Strongly Improved by a Better-Informed Local Scaling of Its Self-Interaction Correction
Source: J Chem Theory Comput. 2026 May 29;22(11):5514–22. doi: 10.1021/acs.jctc.6c00357 (PMC13255254; doi:10.1021/acs.jctc.6c00357)
Supplement: Supplementary file 1 [file ct6c00357_si_001.pdf]

Local Spin Density Approximation Strongly Improved by a Better-Informed Local Scaling of its Self-Interaction Correction: Supplementary Information

Chandra Shahi,[1,a] Rohan Maniar,[1] Jinliang Ning,[2] Raj K. Sah,[2] Mark R. Pederson,[3] Adrienn Ruzsinszky,[1] Juan E. Peralta,[4] Koblar A. Jackson,[4] and John P. Perdew[1]

[1] *Department of Physics and Engineering Physics, Tulane University, New Orleans, Louisiana 70118, USA*

[2] *Department of Physics, Temple University, Philadelphia, Pennsylvania 19122, USA*

[3] *Department of Physics, University of Texas at El Paso, Texas 79968, USA*

[4] *Department of Physics, Central Michigan University, Mount Pleasant, Michigan 48859, USA*

(Dated: 12 May 2026)

[a] Electronic mail: cshahi1@tulane.edu.

TABLE S1. Exchange–correlation energies (in Hartrees) and absolute percentage errors (APEs) for rare gas atoms. Values in the parentheses are corresponding r^{2}SCAN results.[1] Reference values are taken from Refs. 2–4.

System	$E_{\mathrm{xc}}^{\mathrm{ref}}$	$E_{\mathrm{xc}}^{\mathrm{LSIC}-\alpha}$	APE (%)
Ne	−12.499	−12.547(−12.491)	0.39(0.04)
Ar	−30.913	−30.903(−30.879)	0.03(0.07)
Kr	−95.740	−95.672(−95.590)	0.07(0.10)
Xe	−182.200	−181.650(−181.745)	0.30(0.18)

TABLE S2. Interaction energies (in Hartrees) and absolute percentage errors (APEs) for the compressed argon dimer at bond lengths of 1.6 Å, 1.8 Å, and 2.0 Å. Values in parentheses are corresponding r^{2}SCAN values.[1] Reference values are taken from Ref. 5.

System	$E_{\mathrm{int}}^{\mathrm{ref}}$	$E_{\mathrm{int}}^{\mathrm{LSIC}\text{-}\alpha}$	APE (%)
Ar_2 (1.6 Å)	0.58214	0.58396(0.57771)	0.31(0.78)
Ar_2 (1.8 Å)	0.31594	0.31527(0.31571)	0.21(0.07)
Ar_2 (2.0 Å)	0.16449	0.16460(0.16612)	0.07(1.00)

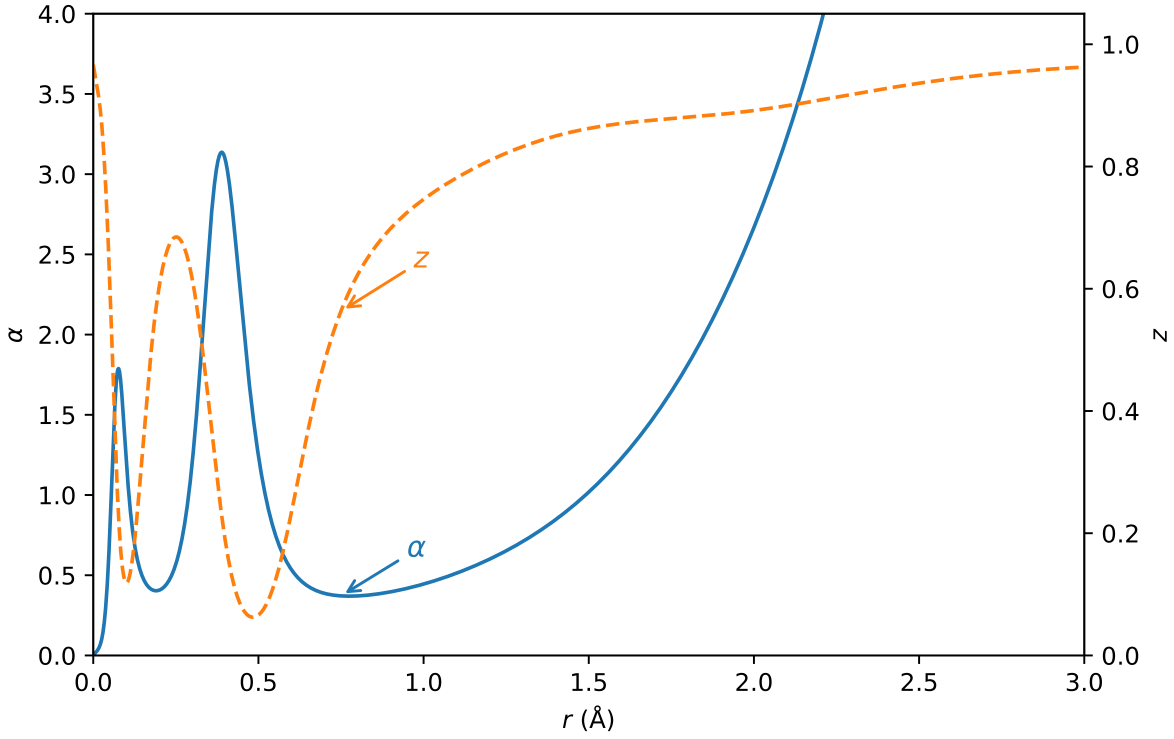

FIG. S1. Iso-orbital indicators α and z for the Ar atom plotted along the radial direction. $r = 0$ is the nuculear position.

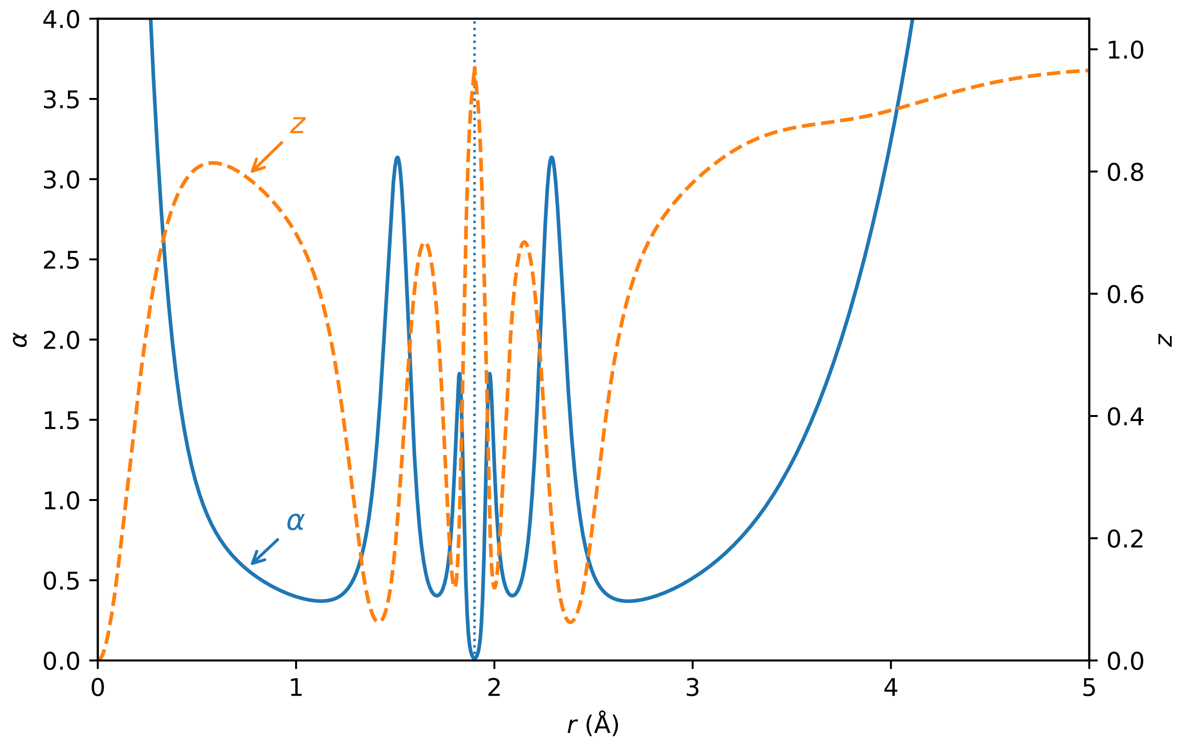

FIG. S2. Iso-orbital indicators α and z plotted along the internuclear axis of the Ar_2 dimer at an interatomic separation of 3.8 Å. The vertical dotted line marks the position of the Ar nucleus. $r = 0$ is the bond center.

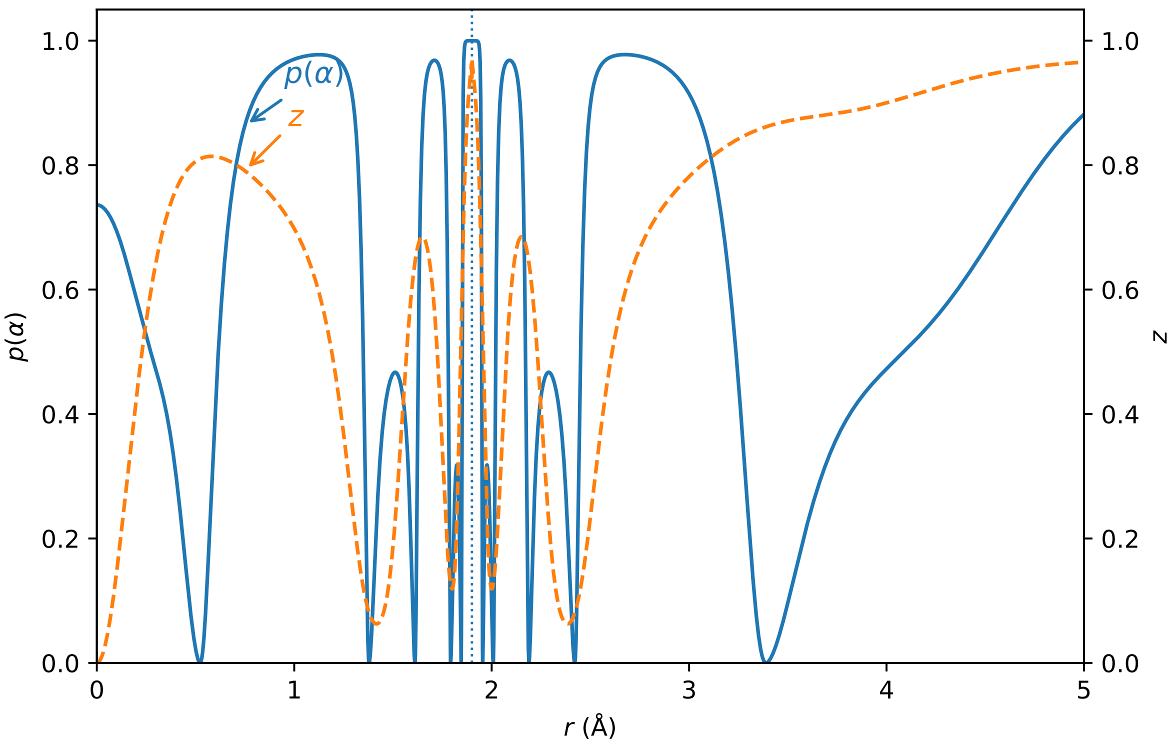

FIG. S3. Scale-down functions for LSIC and LSIC-α in the Ar_2 dimer plotted along the internuclear axis using the self-consistent SCAN density. The LSIC scale-down factor is z while the LSIC-α scale-down factor is $p(\alpha)$. The comparison highlights the different behavior of the two scaling factors in the weak density-overlap region around $r = 0$. The vertical dotted line marks the position of the Ar nucleus.

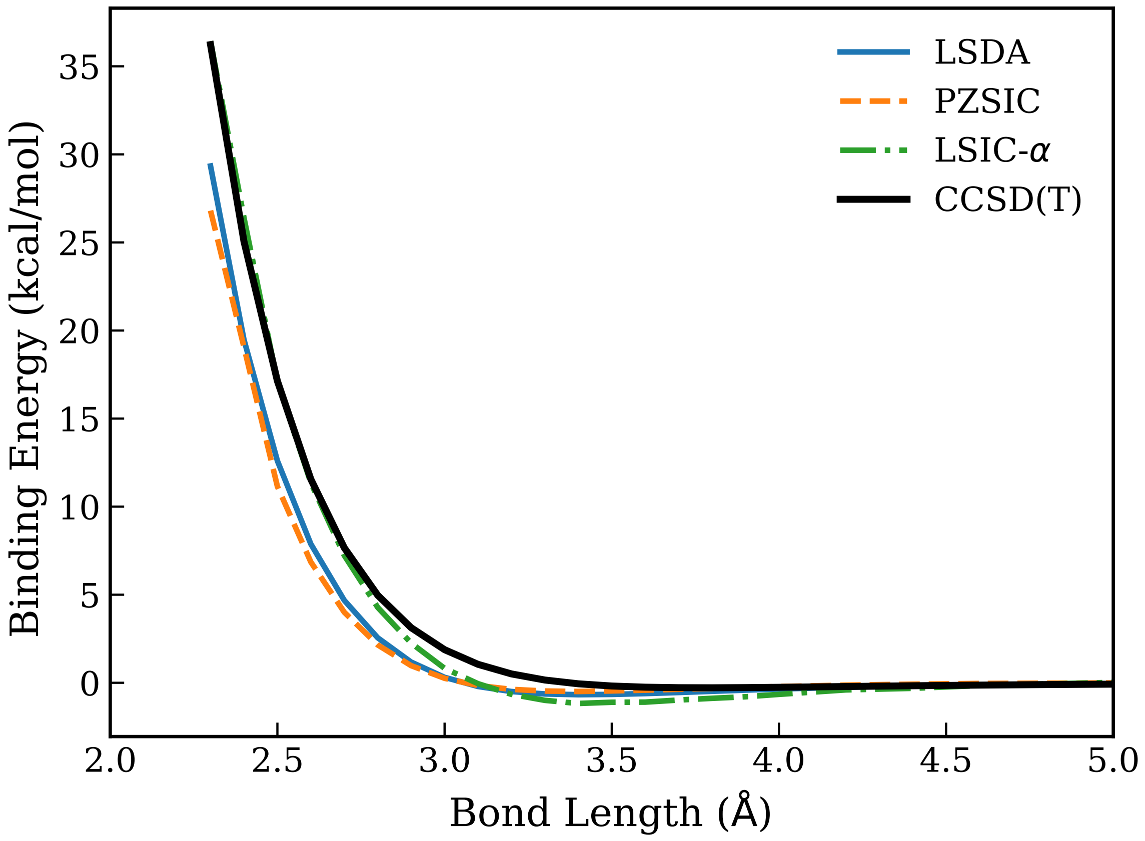

FIG. S4. Binding energy curve of Ar_2 as a function of interatomic distance computed using LSDA, PZSIC, and LSIC-α, compared with the CCSD(T)/CBS reference. The CCSD(T) data were obtained using the PySCF package.[6]

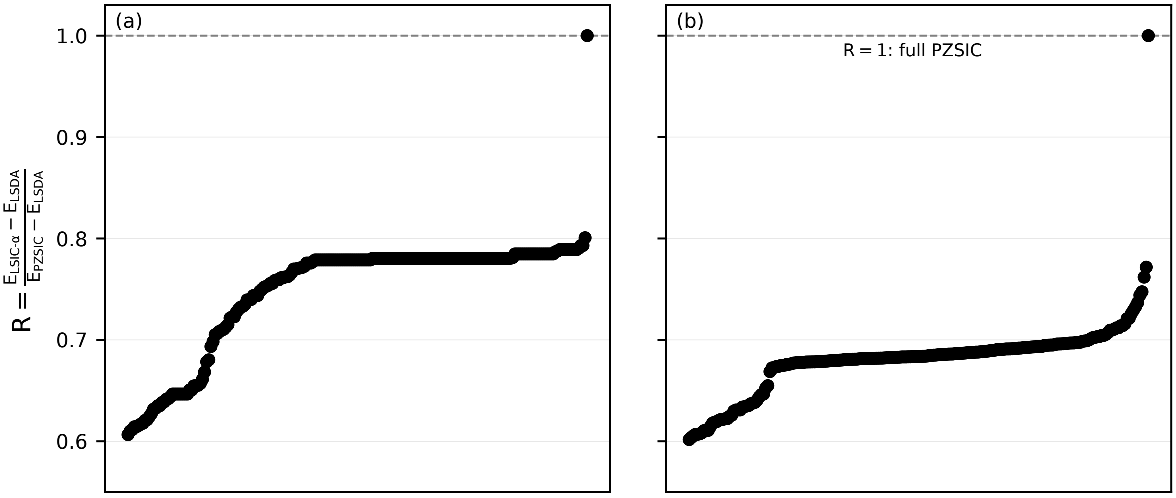

FIG. S5. Fraction of the PZSIC total-energy correction retained by LSIC-α for (a) atoms and (b) molecules of the MGAE109 dataset, defined as $R = (E_{\text{LSIC-}\alpha} - E_{\text{LSDA}})/(E_{\text{PZSIC}} - E_{\text{LSDA}})$. Values near unity indicate retention of the full PZSIC correction, while smaller values indicate stronger local scaling. Systems are ordered by increasing R. LSIC-α retains a larger fraction of the PZSIC correction for atoms than for molecules mainly due to differences in the valence regions; in the deep core (1s), $\alpha \approx 0$ and $f(\alpha) \approx 1$, so nearly the full PZSIC correction is retained for both atoms and molecules.

TABLE S3: Atomization energies (kcal/mol) for the MGAE109 dataset.

#	Molecule	Ref.	LSDA	LSDA-PZSIC	LSIC	LSIC-α	PBE	r^{2}SCAN
1	CH($^2\Pi$)	84.23	92.21	94.98	87.65	84.13	84.47	81.27
2	$CH_2(^3B_1)$	190.75	212.36	211.42	198.96	195.73	194.35	196.74
3	$CH_2(^1A_1)$	181.46	198.51	200.54	186.61	182.69	178.68	174.82
4	$CH_3(^2A_2'')$	307.88	338.62	337.64	316.49	311.15	309.67	311.34
5	CH_4	420.43	461.74	466.89	433.31	424.69	419.77	419.22
6	NH	83.10	95.16	96.64	89.37	83.31	88.26	84.11
7	NH_2	182.59	207.45	208.12	192.23	185.21	188.21	183.24
8	NH_3	298.02	336.64	334.05	309.29	300.68	301.50	294.14
9	OH	107.22	123.94	115.58	110.76	110.39	109.88	108.58

Continued on next page

#	Molecule	Ref.	LSDA	LSDA-PZSIC	LSIC	LSIC-α	PBE	r^2SCAN
10	H_2O	232.98	266.19	247.54	237.71	236.79	234.44	230.37
11	HF	141.63	162.02	143.85	142.35	142.43	142.30	138.81
12	$SiH_2(^1A_1)$	152.22	165.79	172.80	155.68	156.60	147.38	148.55
13	$SiH_2(^3B_1)$	131.48	146.62	151.73	138.25	138.80	131.23	137.88
14	SiH_3	228.01	246.11	258.21	234.06	235.86	221.80	229.46
15	SiH_4	324.95	346.45	364.29	329.87	333.53	312.77	321.47
16	PH_2	153.20	172.90	183.27	162.90	163.22	153.56	154.86
17	PH_3	242.27	268.56	280.74	250.65	250.39	237.98	239.20
18	H_2S	183.91	206.67	206.82	188.64	187.69	181.83	182.51
19	HCl	107.50	121.33	117.14	110.05	109.24	106.66	106.73
20	C_2H_2	405.53	458.86	435.23	408.58	402.44	413.93	401.74
21	CH_2CH_2	563.69	631.30	625.71	576.85	566.25	570.83	562.25
22	CH_3CH_3	712.98	792.91	800.22	727.63	720.96	716.24	711.84
23	CN	181.36	218.23	175.20	167.64	160.92	196.04	176.84
24	HCN	313.43	359.66	331.11	311.27	303.00	325.08	306.93
25	CO	259.74	297.99	256.65	247.26	244.11	268.09	254.72
26	HCO	279.43	331.82	294.32	275.86	271.87	294.43	281.95
27	H_2CO	374.67	432.57	405.42	375.22	371.42	385.19	373.08
28	CH_3OH	513.54	585.28	571.38	519.25	521.15	519.61	511.87
29	N_2	228.48	266.05	235.94	221.00	212.71	241.88	218.18
30	NH_2NH_2	438.60	514.39	509.31	450.11	442.56	451.99	432.89
31	NO	152.75	196.79	158.30	143.72	136.83	170.61	150.73
32	O_2	120.83	173.54	108.09	91.86	94.91	142.60	129.00
33	HOOH	269.03	334.27	297.61	269.91	273.21	282.00	268.73
34	F_2	39.03	77.16	40.95	31.87	30.44	52.53	39.51
35	CO_2	390.16	470.58	376.19	356.15	359.71	414.05	393.18
36	Si_2 (mult=3)	76.38	92.29	82.73	75.16	75.83	78.21	74.91
37	P_2	117.59	141.42	122.80	115.36	115.83	118.98	110.10
38	S_2	104.25	133.51	109.39	98.25	101.98	111.91	108.12

Continued on next page

#	Molecule	Ref.	LSDA	LSDA-PZSIC	LSIC	LSIC-α	PBE	r^2SCAN
39	Cl_2	59.75	81.36	71.62	55.92	60.91	62.51	57.39
40	SiO (mult=1)	193.06	222.59	181.32	175.92	172.05	194.83	186.60
41	SC	171.76	201.45	159.68	159.73	152.87	178.21	167.46
42	SO (mult=3)	126.48	163.87	124.54	113.36	116.27	136.80	128.89
43	ClO	65.45	100.97	76.57	57.90	62.26	77.97	67.87
44	ClF	62.79	93.05	70.65	54.90	59.67	69.75	60.76
45	Si_2H_6	535.89	578.06	606.09	540.57	549.59	518.63	531.99
46	CH_3Cl	396.44	446.91	446.39	402.70	402.94	399.27	395.44
47	CH_3SH	474.49	535.73	538.79	484.45	484.83	477.26	474.09
48	HOCl	166.24	209.57	186.49	163.35	168.53	173.50	164.39
49	SO_2	260.63	323.21	246.61	222.99	229.50	267.57	250.42
50	$AlCl_3$	312.64	343.36	312.89	286.79	302.84	303.58	311.98
51	AlF_3	430.95	479.38	410.22	392.68	412.24	424.44	424.14
52	BCl_3	325.45	382.39	351.39	305.03	324.44	333.68	331.26
53	BF_3	470.96	546.33	481.16	435.13	464.80	480.06	472.43
54	C_2Cl_4	469.82	586.89	538.61	440.94	472.08	495.24	474.34
55	C_2F_4	591.06	740.54	644.07	544.95	582.50	627.23	595.44
56	C_3H_4 (propyne)	705.06	799.96	775.99	709.80	703.62	719.54	703.40
57	C_4H_4O	994.33	1162.29	1105.38	980.36	986.72	1029.07	999.44
58	C_4H_4S	963.65	1117.78	1076.06	954.70	959.32	992.66	968.08
59	C_4H_5N	1071.93	1246.92	1195.89	1063.67	1061.18	1108.32	1075.13
60	C_4H_6 (trans-1,3-butadiene)	1012.73	1148.56	1129.49	1024.53	1014.03	1032.97	1013.32
61	C_4H_6 (2-butyne)	1004.49	1139.50	1115.77	1009.75	1003.97	1023.69	1003.47
62	C_5H_5N	1238.14	1437.33	1373.14	1222.55	1216.20	1282.27	1243.09
63	CCH	265.31	308.31	280.13	263.02	259.60	276.08	265.67
64	CCl_4	316.19	400.48	370.14	291.56	326.07	329.68	316.73
65	CF_3CN	641.17	785.36	693.76	597.42	631.43	676.84	639.91
66	CF_4	477.93	594.44	518.46	433.03	482.20	499.61	480.54
67	CH_2OH	410.08	476.38	453.92	412.56	415.13	421.11	413.75

Continued on next page

#	Molecule	Ref.	LSDA	LSDA-PZSIC	LSIC	LSIC-α	PBE	r^2SCAN
68	CH_3CN	616.02	703.93	675.34	615.94	607.35	633.89	612.06
69	CH_3NH_2	582.31	659.75	660.79	594.83	588.92	590.09	578.75
70	CH_3NO_2	601.82	740.69	658.16	579.16	585.12	639.36	607.17
71	$CHCl_3$	345.79	419.00	399.26	333.12	354.71	356.62	346.50
72	CHF_3	458.73	556.50	500.95	431.34	462.39	476.57	460.62
73	ClF_3	127.31	219.56	138.66	97.20	114.03	155.39	133.42
74	H_2	109.49	112.85	114.52	114.52	114.52	104.57	107.60
75	CH_2CH	446.09	506.11	495.10	455.72	447.55	457.11	449.71
76	$HCOOCH_3$	785.90	919.39	866.24	772.61	784.88	809.62	788.68
77	HCOOH	501.53	595.91	539.78	489.79	497.89	521.30	503.27
78	NF_3	205.67	309.21	238.11	181.87	199.05	242.03	212.89
79	PF_3	365.01	432.73	375.47	322.37	355.65	365.14	353.97
80	SH	87.00	99.42	100.52	91.30	91.70	87.75	88.59
81	$SiCl_4$	388.73	439.74	409.79	352.13	387.73	378.93	384.21
82	SiF_4	576.30	646.97	569.25	514.88	562.40	563.47	563.15
83	C_2H_5	603.93	676.13	673.12	613.44	607.53	611.37	608.54
84	C_4H_6 (bicyclobutane)	987.56	1132.47	1114.83	995.52	996.39	1010.21	989.82
85	C_4H_6 (cyclobutene)	1001.97	1142.80	1131.96	1011.14	1008.63	1022.78	1001.52
86	HCOCOH	633.99	750.52	689.38	623.45	624.63	661.62	635.06
87	CH_3CHO	677.44	777.05	749.12	678.77	676.87	693.46	678.10
88	C_2H_4O	651.11	754.92	730.94	653.24	655.90	668.73	652.91
89	C_2H_5O	699.05	798.96	790.30	703.26	706.94	709.64	699.56
90	CH_3OCH_3	798.46	910.03	899.36	802.98	808.94	808.89	797.95
91	CH_3CH_2OH	810.77	922.25	909.59	816.84	822.38	820.71	809.82
92	C_3H_4 (allene)	703.47	803.13	779.21	714.53	702.62	722.44	705.70
93	C_3H_4 (cyclopropene)	683.01	783.13	764.67	692.52	685.77	700.46	682.85
94	CH_3COOH	803.68	937.50	882.85	791.32	803.39	826.63	806.08
95	CH_3COCH_3	978.46	1118.72	1091.28	979.55	981.00	998.71	980.66
96	C_3H_6 (cyclopropane)	853.68	966.73	963.79	866.90	863.53	867.24	855.01

Continued on next page

#	Molecule	Ref.	LSDA	LSDA-PZSIC	LSIC	LSIC-α	PBE	r^2SCAN
97	CH_3CHCH_2	860.88	968.94	963.58	874.11	865.57	872.63	860.49
98	C_3H_8	1007.14	1126.52	1135.35	1022.07	1018.79	1014.18	1006.46
99	$C_2H_5OCH_3$	1095.62	1246.94	1237.50	1100.62	1110.12	1109.94	1095.87
100	C_4H_{10} (isobutane)	1303.40	1461.81	1471.60	1316.11	1317.49	1312.85	1302.35
101	C_4H_{10} (antiperiplanar butane)	1301.68	1460.13	1470.42	1316.60	1316.70	1312.09	1301.07
102	C_4H_8 (cyclobutane)	1149.37	1302.21	1307.01	1161.97	1165.00	1166.22	1149.85
103	C_4H_8 (isobutene)	1158.97	1306.86	1301.95	1170.92	1166.10	1174.10	1158.94
104	C_5H_8 (spiropentane)	1284.73	1471.30	1458.38	1297.23	1299.82	1313.51	1289.53
105	C_6H_6	1368.10	1570.43	1512.20	1356.96	1350.35	1406.95	1374.72
106	CH_3CO	581.99	675.54	637.73	579.74	576.37	602.60	586.58
107	$(CH_3)_2CH$	901.02	1014.36	1015.64	913.70	910.09	913.43	906.68
108	$(CH_3)_3C$	1199.70	1352.81	1355.38	1211.30	1210.70	1215.33	1205.17
109	H_2CCO	532.73	625.34	569.40	524.73	520.65	556.38	537.31

TABLE S4: Forward (F) and reverse (R) barrier heights (in kcal/mol) for the BH76 dataset.

#	Reaction	Dir	Ref	LSDA	LSDA-PZSIC	LSIC	LSIC-α	PBE	r^2SCAN
1	$H + N_2O \rightarrow OH + N_2$	F	17.7	2.4	3.5	18.1	21.1	9.6	10.0
		R	82.6	34.2	84.8	105.6	104.7	54.2	64.6
2	$H + HF \rightarrow HF + H$	F	42.1	18.4	52.2	49.4	57.8	27.0	29.0
		R	42.1	18.4	52.2	49.4	57.8	27.0	29.0
3	$H + HCl \rightarrow HCl + H$	F	17.8	3.0	19.0	20.6	24.0	10.3	10.0
		R	17.8	3.0	19.0	20.6	24.0	10.3	10.0
4	$H + CH_3F \rightarrow HF + CH_3$	F	30.5	12.8	43.8	34.3	43.1	18.0	18.9
		R	56.9	31.9	56.9	68.0	68.6	41.1	46.9
5	$H + F_2 \rightarrow HF + F$	F	1.5	-15.9	1.6	7.3	7.3	-9.8	-9.6
		R	104.8	68.9	104.6	117.2	118.8	79.9	89.7

Continued on next page

#	Reaction	Dir	Ref	LSDA	LSDA-PZSIC	LSIC	LSIC-α	PBE	r^2SCAN
6	CH_3 + ClF → CH_3F + Cl	F	7.1	-12.4	8.6	10.9	9.6	-7.2	-4.2
		R	59.8	40.1	68.9	64.4	66.8	40.8	45.7
7	F^- + CH_3F → CH_3F + F^-	F	-0.6	-12.3	2.0	6.0	0.8	-8.4	-7.5
		R	-0.6	-12.3	2.0	6.0	0.8	-8.4	-7.5
8	fch3fcomp → fch3fcomp	F	13.4	5.9	17.8	14.6	16.5	6.4	7.9
		R	13.4	5.9	17.8	14.6	16.5	6.4	7.9
9	Cl^- + CH_3Cl → CH_3Cl + Cl^-	F	2.5	-8.1	6.0	7.1	4.9	-5.3	-4.6
		R	2.5	-8.1	6.0	7.1	4.9	-5.3	-4.6
10	clch3clcomp → clch3clcomp	F	13.5	5.7	17.8	13.4	15.5	6.1	7.1
		R	13.5	5.7	17.8	13.4	15.5	6.1	7.1
11	F^- + CH_3Cl → Cl^- + CH_3F	F	-12.3	-23.5	-12.2	-6.7	-13.4	-19.5	-20.4
		R	19.8	9.0	25.8	25.1	25.2	11.2	14.5
12	fch3clcomp1 → fch3clcomp2	F	3.5	-1.6	5.6	3.9	4.5	-1.3	-1.3
		R	29.6	20.8	36.4	30.3	34.5	20.7	24.2
13	OH^- + CH_3F → CH_3OH + F^-	F	-2.7	-15.6	-1.3	2.7	-1.0	-11.9	-10.4
		R	17.6	6.4	22.7	25.6	21.1	9.9	10.9
14	hoch3fcomp2 → hoch3fcomp1	F	11.0	1.8	14.7	11.4	13.9	2.2	4.3
		R	47.7	47.3	59.5	50.3	53.7	43.1	44.6
15	H + N_2 → HN_2	F	14.6	-2.2	9.2	16.3	18.6	5.2	4.6
		R	10.9	9.4	27.6	21.1	20.3	9.0	9.3
16	H + CO → HCO	F	3.2	-7.6	-1.0	3.3	3.1	-1.7	-3.3
		R	22.8	26.3	36.9	32.0	31.1	24.6	23.9
17	H + C_2H_4 → C_2H_5	F	2.0	-5.3	-0.7	6.4	1.6	-0.2	-3.2
		R	42.0	39.5	50.1	44.7	45.3	40.4	43.1
18	CH_3 + C_2H_4 → C_3H_7	F	6.4	-5.7	-1.1	13.9	4.6	1.5	2.5
		R	33.0	33.1	46.6	30.9	35.2	29.8	31.3
19	HCN → HNC	F	48.1	44.6	50.9	52.5	54.1	45.4	46.0
		R	33.0	30.4	38.6	38.1	40.2	30.3	31.5
20	H + HCl → H_2 + Cl	F	6.1	-3.4	3.3	4.6	7.5	0.4	-0.2

Continued on next page

#	Reaction	Dir	Ref	LSDA	LSDA-PZSIC	LSIC	LSIC-α	PBE	r^2SCAN
		R	8.0	-9.2	0.9	9.1	13.0	-3.1	0.6
21	$OH + H_2 \rightarrow H_2O + H$	F	5.2	-18.2	0.0	8.3	11.8	-6.4	-1.6
		R	21.6	11.2	17.3	20.9	24.1	13.6	12.6
22	$CH_3 + H_2 \rightarrow CH_4 + H$	F	11.9	-5.3	-0.3	11.5	14.7	3.8	7.6
		R	15.0	4.9	14.4	13.8	13.8	9.3	7.9
23	$OH + CH_4 \rightarrow H_2O + CH_3$	F	6.3	-16.9	4.4	9.0	9.7	-5.5	-1.0
		R	19.5	2.2	7.0	19.4	22.9	8.9	12.9
24	$H + H_2 \rightarrow H + H_2$	F	9.7	-2.6	5.6	8.7	11.7	3.7	2.5
		R	9.7	-2.6	5.6	8.7	11.7	3.7	2.5
25	$OH + NH_3 \rightarrow H_2O + NH_2$	F	3.4	-23.8	4.0	7.2	8.0	-12.0	-6.9
		R	13.7	-10.7	10.2	16.6	18.8	-0.7	4.0
26	$HCl + CH_3 \rightarrow Cl + CH_4$	F	1.8	-13.6	-7.8	0.7	0.9	-5.9	-2.7
		R	6.8	-9.2	4.6	7.5	5.5	-3.9	-1.6
27	$OH + C_2H_6 \rightarrow H_2O + C_2H_5$	F	3.5	-20.7	1.9	7.2	7.5	-8.9	-4.3
		R	20.4	4.8	10.1	22.0	23.4	10.8	14.2
28	$F + H_2 \rightarrow HF + H$	F	1.6	-23.9	-3.3	4.0	5.4	-13.0	-7.3
		R	33.8	25.2	26.0	31.9	33.4	24.8	23.9
29	$O + CH_4 \rightarrow OH + CH_3$	F	14.4	-10.0	12.9	16.1	15.6	-0.3	2.9
		R	8.9	-9.2	-0.5	9.4	11.8	-0.5	3.6
30	$H + PH_3 \rightarrow H_2 + PH_2$	F	2.9	-7.3	-0.1	1.5	2.9	-1.8	-3.2
		R	24.7	9.9	17.6	28.8	31.0	18.4	20.1
31	$H + OH \rightarrow H_2 + O$	F	10.9	-1.9	9.8	10.1	13.1	3.6	3.1
		R	13.2	-12.9	8.5	14.5	17.8	-1.7	2.1
32	$H + H_2S \rightarrow H_2 + HS$	F	3.9	-6.7	1.1	2.0	3.2	-1.2	-2.5
		R	17.2	0.1	9.8	19.4	22.1	9.3	11.1
33	$O + HCl \rightarrow OH + Cl$	F	10.4	-23.3	9.0	12.5	13.2	-10.8	-4.1
		R	9.9	-18.0	7.9	12.7	13.9	-9.0	-2.3
34	$NH_2 + CH_3 \rightarrow NH + CH_4$	F	8.9	-8.4	2.0	9.8	10.8	0.7	4.6
		R	22.0	2.4	19.4	24.4	23.0	10.8	13.4

Continued on next page

#	Reaction	Dir	Ref	LSDA	LSDA-PZSIC	LSIC	LSIC-α	PBE	r^2SCAN
35	$NH_2 + C_2H_5 \rightarrow NH + C_2H_6$	F	9.8	-5.8	4.7	12.6	11.7	2.9	6.1
		R	19.4	-1.3	16.6	22.8	21.2	7.8	10.3
36	$C_2H_6 + NH_2 \rightarrow C_2H_5 + NH_3$	F	11.3	-9.5	9.1	14.7	14.4	1.6	5.4
		R	17.8	2.9	11.1	20.1	19.5	10.0	13.0
37	$NH_2 + CH_4 \rightarrow NH_3 + CH_3$	F	13.9	-6.0	11.3	16.4	16.2	4.5	8.4
		R	16.9	0.1	7.7	17.4	18.6	7.7	11.5
38	$C_5H_8 \rightarrow C_5H_8$	F	39.7	24.9	64.4	62.3	54.5	31.2	33.0
		R	39.7	24.9	64.4	62.3	54.5	31.2	33.0

TABLE S5: Adiabatic ionization potentials (kcal/mol) for the G21IP dataset.

#	System → Cation	Ref	LSDA	LSDA-PZSIC	LSIC	LSIC-α	PBE	r^2SCAN
1	$H \rightarrow H^+$	314.9	300.4	313.7	313.7	313.7	300.4	313.8
2	$Li \rightarrow Li^+$	123.3	126.2	127.7	122.4	119.1	128.9	124.3
3	$Be \rightarrow Be^+$	214.9	208.2	211.9	205.1	200.3	207.6	202.7
4	$B \rightarrow B^+$	190.4	199.2	207.1	201.7	198.4	200.0	202.1
5	$C \rightarrow C^+$	259.6	269.5	269.1	266.2	266.5	266.1	269.1
6	$N \rightarrow N^+$	335.3	345.9	339.3	339.9	340.3	339.8	341.6
7	$O \rightarrow O^+$	313.8	322.4	329.8	323.4	313.1	323.6	315.4
8	$F \rightarrow F^+$	401.7	414.2	407.7	405.4	403.3	407.3	404.7
9	$Na \rightarrow Na^+$	118.5	123.8	123.6	115.1	116.9	123.6	119.0
10	$Mg \rightarrow Mg^+$	176.3	178.2	179.6	169.8	171.6	175.6	169.9
11	$Al \rightarrow Al^+$	138.0	139.0	143.1	141.7	141.4	140.1	142.3
12	$Si \rightarrow Si^+$	188.0	190.1	188.7	188.8	187.7	187.7	191.8
13	$P \rightarrow P^+$	241.9	243.4	240.1	241.8	239.2	241.9	245.1
14	$S \rightarrow S^+$	239.0	243.4	249.7	245.7	243.4	237.4	238.0
15	$Cl \rightarrow Cl^+$	299.1	305.6	303.9	301.5	299.8	300.0	299.9

Continued on next page

#	System → Cation	Ref	LSDA	LSDA-PZSIC	LSIC	LSIC-α	PBE	r^{2}SCAN
16	$CH_4 \to CH_4^+$	296.3	289.1	317.0	306.2	302.6	287.2	287.5
17	$NH_3 \to NH_3^+$	235.7	242.0	241.9	237.9	233.3	235.4	230.5
18	$OH \to OH^+$	300.9	311.1	307.7	304.0	300.8	305.7	302.2
19	$H_2O \to H_2O^+$	292.6	299.8	292.4	291.2	286.9	291.7	287.4
20	$HF \to HF^+$	371.3	381.4	369.9	371.2	367.9	371.9	367.8
21	$SiH_4 \to SiH_4^+$	255.4	252.0	268.1	256.4	258.0	248.1	250.4
22	$PH \to PH^+$	234.1	236.9	235.8	235.6	232.9	236.2	240.3
23	$PH_2 \to PH_2^+$	226.4	230.2	232.5	229.9	227.5	229.7	234.3
24	$PH_3 \to PH_3^+$	227.8	230.0	229.7	224.3	221.5	225.4	223.1
25	$SH \to SH^+$	239.3	244.2	246.1	242.6	241.2	237.0	238.5
26	$HCl \to HCl^+$	294.5	297.8	295.7	294.9	291.1	294.7	293.2
27	$C_2H_2 \to C_2H_2^+$	264.6	268.1	263.6	260.8	254.9	259.9	257.0
28	$C_2H_4 \to C_2H_4^+$	243.7	245.6	243.0	240.5	232.5	238.5	235.7
29	$CO \to CO^+$	323.0	325.0	337.4	328.9	323.3	320.0	319.2
30	$N_2 \to N_2^+$	359.4	360.8	385.2	383.0	382.0	355.0	359.8
31	$O_2 \to O_2^+$	277.7	284.7	290.3	284.5	284.3	284.3	288.1
32	$P_2 \to P_2^+$	242.9	247.8	241.1	245.2	241.0	242.8	243.1
33	$S_2 \to S_2^+$	215.7	218.1	223.9	219.7	220.2	218.2	222.4
34	$Cl_2 \to Cl_2^+$	265.1	260.1	269.9	264.7	264.9	258.3	261.3
35	$ClF \to ClF^+$	291.7	289.0	298.1	296.1	295.7	287.1	288.5
36	$SC \to SC^+$	261.2	264.3	267.1	270.5	261.4	260.4	258.4

TABLE S6: SIE4×4 dataset: dissociation energies (kcal/mol) as a function of bond length ratio R/R_e.

#	Reaction	R/R_e	Ref	LSDA	LSDA-PZSIC	LSIC	LSIC-α	r^{2}SCAN
1	$H_2^+ \to H + H^+$	1.00	64.4	65.9	64.4	64.4	64.4	67.8
		1.25	58.9	65.3	58.9	58.9	58.9	64.9

Continued on next page

#	Reaction	R/R_e	Ref	LSDA	LSDA-PZSIC	LSIC	LSIC-α	r^2SCAN
		1.50	48.7	59.6	48.7	48.7	48.7	57.8
		1.75	38.3	53.6	38.2	38.2	38.2	50.8
2	$He_2^+ \rightarrow He + He^+$	1.00	56.9	84.5	62.7	55.2	52.8	74.1
		1.25	46.9	82.7	48.8	44.2	42.3	71.3
		1.50	31.3	75.2	30.9	28.3	26.9	63.4
		1.75	19.1	70.2	17.5	16.1	15.1	58.4
3	$(NH_3)^{2+} \rightarrow NH_3 + NH_3^+$	1.00	35.9	56.3	47.7	37.5	40.1	42.6
		1.25	25.9	50.0	33.3	32.6	33.7	37.7
		1.50	13.4	42.3	17.5	21.5	20.7	30.5
		1.75	4.9	38.5	8.5	11.6	10.2	26.7
4	$(H_2O)^{2+} \rightarrow H_2O + H_2O^+$	1.00	39.7	67.0	45.7	40.1	43.4	52.0
		1.25	29.1	61.4	27.8	33.3	33.0	48.2
		1.50	16.9	-16.4	14.4	18.4	17.4	42.2
		1.75	9.3	52.0	8.0	11.1	10.4	39.6

TABLE S7. Reaction energies (kcal/mol) for the SIE11 dataset.

#	Reaction	Ref	LSDA	LSDA-PZSIC	LSIC	LSIC-α	PBE	r^2SCAN
1	$He \rightarrow He^+ \rightarrow He^{2+}$	57.44	84.47	62.73	55.19	52.76	18.94	74.11
2	$NH_3 \rightarrow NH_3^+ \rightarrow NH_3^{2+}$	35.34	56.32	47.43	37.26	40.01	11.73	42.62
3	$H_2O \rightarrow H_2O^+ \rightarrow H_2O^{2+}$	37.25	66.97	45.20	40.49	43.62	19.80	51.97
4	$but^+ + ethyl \rightarrow ethyl^+$	35.28	54.59	46.74	29.18	40.98	7.67	41.13
5	$CH_3 + acetyl \rightarrow aceton^+$	22.57	42.65	61.97	24.46	35.77	8.07	29.28
6	clclf → clfcl	-1.01	29.26	14.93	3.65	-2.85	23.23	16.90
7	$C_2H_4 + F_2 \rightarrow C_2H_4\_F_2$	1.08	3.89	0.99	-1.66	2.10	1.51	2.32
8	Li + bz → bz_Li	9.50	13.90	19.71	-4.01	13.12	-4.23	7.20
9	$NH_3 + ClF \rightarrow NH_3\_ClF$	10.50	23.81	16.02	6.14	9.15	6.66	16.46
10	MgO + Na → NaOMg	69.56	72.63	64.32	70.17	67.47	-2.36	75.24
11	$Li + F_2 \rightarrow Li\_F_2$	94.36	122.68	89.54	93.25	90.29	28.14	123.96

TABLE S8. Reaction energies (kcal/mol) for the DARC dataset.

#	Reaction	Ref	LSDA	LSDA-PZSIC	LSIC	LSIC-α	PBE	r^{2}SCAN
1	ethene + butadiene → P1	-45.4	-62.1	-81.5	-22.7	-54.2	-42.9	-45.9
2	ethine + butadiene → P2	-60.8	-80.7	-103.6	-46.5	-67.5	-62.2	-64.1
3	ethene + cpdiene → P3	-29.9	-42.5	-63.9	-9.1	-41.8	-25.0	-28.2
4	ethine + cpdiene → P4	-33.6	-48.6	-71.8	-21.4	-43.7	-32.2	-33.7
5	ethene + chdiene → P5	-37.6	-51.5	-71.1	-14.2	-47.1	-32.5	-36.5
6	ethine + chdiene → P6	-49.0	-66.3	-86.9	-33.5	-57.1	-47.9	-50.7
7	furane + malein → P7	-14.0	-21.6	-53.7	4.6	-33.1	-4.4	-8.9
8	furane + malein → P7X	-15.9	-23.5	-56.0	1.9	-34.9	-6.5	-10.9
9	furane + maleinNH → P8	-16.8	-24.3	-56.8	1.4	-36.6	-7.0	-11.7
10	furane + maleinNH → P8X	-18.9	-26.6	-60.6	-1.9	-39.1	-9.5	-14.1
11	cpdiene + malein → P9	-31.7	-41.8	-64.2	-6.1	-44.7	-22.4	-27.2
12	cpdiene + malein → P9X	-32.2	-42.7	-64.9	-7.0	-44.7	-23.4	-28.0
13	cpdiene + maleinNH → P10	-34.2	-44.3	-66.9	-8.8	-47.5	-24.8	-29.8
14	cpdiene + maleinNH → P10X	-34.6	-45.1	-68.7	-10.0	-48.0	-25.7	-30.5

TABLE S9. Interaction energies (kcal/mol) for the S22 dataset.

Complex	Ref	LSDA	LSDA-PZSIC	LSIC	LSIC-α	PBE	r^2SCAN
Ammonia dimer	−3.17	−5.17	−4.72	0.52	−2.51	−2.91	−2.99
Water dimer	−5.02	−7.91	−7.60	−1.55	−4.82	−5.03	−5.21
Formic acid dimer	−18.61	−27.11	−26.10	−9.60	−16.47	−18.38	−20.00
Formamide dimer	−15.96	−22.08	−22.90	−9.01	−15.15	−14.94	−15.96
Uracil dimer (H-bonded)	−20.65	−26.38	−26.24	−11.63	−17.95	−18.67	−19.87
2-Pyridoxine–2-aminopyridine complex	−16.71	−23.02	−22.17	−7.67	−13.43	−15.51	−16.34
Adenine–thymine (Watson–Crick)	−16.37	−22.21	−20.68	−14.04	−7.92	−14.51	−15.45
Methane dimer	−0.53	−0.83	−0.60	1.72	−0.52	−0.09	−0.30
Ethene dimer	−1.51	−2.49	−2.12	3.77	−0.36	−0.35	−0.89
Benzene–methane complex	−1.50	−2.03	−1.80	3.45	−0.93	−0.07	−0.70
Benzene dimer (parallel)	−2.73	−2.57	−2.07	13.93	−9.34	1.85	−0.57
Pyrazine dimer	−4.42	−4.42	−3.35	12.90	−10.09	0.67	−2.12
Uracil dimer (stacked)	−10.12	−10.22	−8.54	7.84	−11.55	−2.83	−7.41
Indole–benzene complex	−5.22	−4.35	−3.19	16.51	−10.11	2.15	−1.43
Adenine–thymine complex	−12.23	−12.03	−9.76	12.25	−10.51	11.74	−7.74
Ethene–ethyne complex	−1.53	−2.29	−2.25	1.04	−0.61	−1.20	−1.26
Benzene–water complex	−3.28	−4.54	−4.52	1.39	−4.64	−2.13	−3.14
Benzene–ammonia complex	−2.35	−3.12	−3.04	2.35	−3.04	−1.02	−1.86
Benzene–HCN complex	−4.46	−5.92	−6.61	1.03	−4.53	−2.93	−3.88
Benzene dimer (T-shaped)	−2.74	−3.09	−2.86	5.17	−2.70	−0.19	−1.25
Indole–benzene (T-shaped)	−5.73	−6.40	−5.75	6.13	−6.57	−2.19	−3.83
Phenol dimer	−7.05	−9.11	−8.86	2.57	−5.48	−4.00	−5.51

TABLE S10. Interaction energies (kcal/mol) for the B30 dataset.

#	Complex	Ref	LSDA	LSDA-PZSIC	LSIC	LSIC-α	PBE	r^2SCAN
1	$Cl^- \cdots ClF$	-43.91	-62.70	-41.19	-30.12	-34.72	-53.04	-52.88
2	$Br^- \cdots ClF$	-42.61	-61.91	-55.67	-40.69	-44.98	-52.69	-52.24
3	$Cl^- \cdots BrF$	-46.42	-62.27	-61.03	-43.88	-52.30	-52.84	-53.02
4	$Br^- \cdots BrF$	-43.67	-59.98	-58.30	-42.47	-48.37	-51.00	-50.87
5	$NH_3 \cdots ClF$	-12.10	-24.62	-19.42	-7.02	-11.19	-17.38	-17.28
6	$NH_3 \cdots BrF$	-16.03	-28.21	-24.41	-11.41	-16.61	-20.34	-20.54
7	$Cl^- \cdots SF_2$	-31.52	-43.97	-44.21	-26.55	-35.71	-35.39	-35.63
8	$Br^- \cdots SF_2$	-25.80	-38.20	-38.13	-21.96	-28.99	-30.53	-30.37
9	$Cl^- \cdots SeF_2$	-40.96	-52.20	-53.54	-35.61	-46.20	-43.17	-43.99
10	$Br^- \cdots SeF_2$	-35.50	-47.13	-47.50	-31.26	-39.09	-38.81	-39.17
11	$NH_3 \cdots SF_2$	-7.98	-14.77	-13.64	-2.90	-9.58	-8.96	-9.69
12	$NH_3 \cdots SeF_2$	-13.23	-21.30	-16.94	-5.20	-13.18	-13.90	-15.10
13	$Cl^- \cdots SCF_2$	-9.54	-17.63	-15.64	-5.95	-10.76	-12.92	-11.90
14	$Br^- \cdots SCF_2$	-7.80	-15.23	-13.82	-4.82	-9.48	-11.08	-10.01
15	$Cl^- \cdots SeCF_2$	-13.61	-22.62	-28.78	-13.89	-17.76	-17.39	-16.25
16	$Br^- \cdots SeCF_2$	-11.22	-19.66	-22.87	-10.78	-13.98	-14.99	-13.78
17	$NH_3 \cdots SCF_2$	-1.67	-2.69	-3.06	1.35	-0.74	-1.14	-1.13
18	$NH_3 \cdots SeCF_2$	-2.73	-4.23	-4.84	0.65	-1.20	-2.23	-2.21
19	$Cl^- \cdots SPF_3$	-8.42	-15.30	-19.82	-5.49	-11.51	-11.12	-9.84
20	$Br^- \cdots SPF_3$	-6.76	-12.63	-19.07	-4.42	-10.44	-8.99	-7.79
21	$Cl^- \cdots SePF_3$	-15.45	-26.01	-22.93	-12.83	-15.59	-20.39	-18.37
22	$Br^- \cdots SePF_3$	-12.48	-21.89	-17.69	-9.55	-11.45	-16.96	-14.99
23	$NH_3 \cdots SPF_3$	-1.42	-2.85	-12.28	-0.42	-4.98	-1.24	-1.10
24	$NH_3 \cdots SePF_3$	-2.84	-5.18	-5.60	0.24	-1.73	-2.86	-2.64
25	$Cl^- \cdots PF_3$	-21.37	-29.50	-32.35	-15.45	-26.62	-22.52	-23.21
26	$Br^- \cdots PF_3$	-15.73	-22.93	-21.95	-9.47	-17.71	-17.28	-17.58
27	$Cl^- \cdots AsF_3$	-34.25	-41.97	-46.06	-27.33	-40.64	-33.90	-35.25
28	$Br^- \cdots AsF_3$	-27.42	-35.28	-38.83	-22.06	-32.61	-28.28	-29.03
29	$NH_3 \cdots PF_3$	-4.85	-8.06	-8.94	1.14	-5.80	-3.98	-5.04
30	$NH_3 \cdots AsF_3$	-9.31	-13.96	-15.67	-1.89	-11.04	-8.03	-9.61

THREE-PARAMETER SCALE FACTOR $p(a, b, c, \alpha_\sigma)$

Eq (8) can be smoothly generalized by adding $c\alpha_\sigma^3$ $(c > 0)$ to the numerator and denominator of the outermost exponent, as shown in Figure S6. The third parameter c is not needed to correct LSDA, but might be useful for the correction of other functionals.

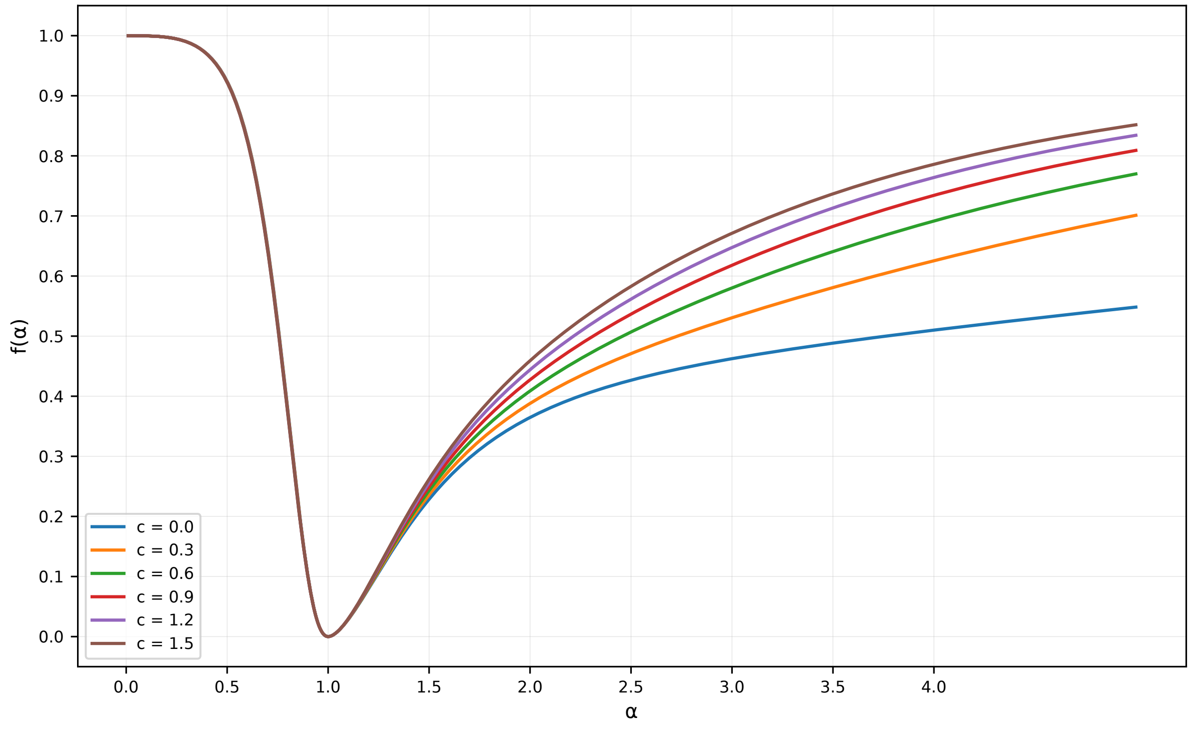

FIG. S6. Scale-down function for different values of c. The case $c = 0$ corresponds to the LSIC-α form used in this work.

REFERENCES

[1] J. W. Furness, A. D. Kaplan, J. Ning, J. P. Perdew, and J. Sun, J. Chem. Phys. **156** (2022).

[2] A. D. Becke, Phys. Rev. A **38**, 3098 (1988).

[3] S. J. Chakravorty, S. R. Gwaltney, E. R. Davidson, F. A. Parpia, and C. F. p Fischer, Phys. Rev. A **47**, 3649 (1993).

[4] S. P. McCarthy and A. J. Thakkar, J. Chem. Phys. **134**, 044102 (2011).

[5] K. Patkowski, G. Murdachaew, C.-M. Fou, and K. Szalewicz, Mol. Phys. **103**, 2031 (2005).

[6] Q. Sun, T. C. Berkelbach, N. S. Blunt, G. H. Booth, S. Guo, Z. Li, J. Liu, J. D. McClain, E. R. Sayfutyarova, S. Sharma, *et al.*, WIREs Comput Mol Sci. **8**, e1340 (2018).
